# Evaluation of the Immunosafety of Cucurbit[n]uril on Peripheral Blood Mononuclear Cells In Vitro

**DOI:** 10.3390/molecules25153388

**Published:** 2020-07-27

**Authors:** Ekaterina Pashkina, Alina Aktanova, Elena Blinova, Irina Mirzaeva, Ekaterina Kovalenko, Nadezhda Knauer, Aleksandr Ermakov, Vladimir Kozlov

**Affiliations:** 1 Research Institute of Fundamental and Clinical Immunology, 14, Yadrintsevskaya st., 630099 Novosibirsk, Russia; aktanova_al@mail.ru (A.A.); blinovaelena-85@yandex.ru (E.B.); knauern@gmail.com (N.K.); niiki01@online.nsk.su (V.K.); 2 Novosibirsk State Medical University, 52, Krasny Prospect, 630091 Novosibirsk, Russia; aleermak@mail.ru; 3 Institute of Chemical Biology and Fundamental Medicine SB RAS, 8, Lavrentiev ave., 630090 Novosibirsk, Russia; 4 Nikolaev Institute of Inorganic Chemistry SB RAS, 3, Lavrentiev ave., 630090 Novosibirsk, Russia; dairdre@gmail.com (I.M.); e.a.kovalenko@niic.nsc.ru (E.K.)

**Keywords:** nanoparticles, cucurbiturils, immunosafety, immune cells

## Abstract

Cucurbiturils (CB[n]s) are nanoscale macrocyclic compounds capable of encapsulating a molecule or part of a molecule by forming host–guest complexes. Integration of drugs with CB[n] is used for the following purposes: controlling clearance; protection of the drug from biodegradation; targeted delivery to specific organs, tissues, or cells; reduction of toxicity; and improving solubility. One of the major problems encountered in the application of new drug delivery systems is lack of knowledge of their biological properties. CB[n], unlike many other often toxic nanoparticles, has extremely low toxicity, even at high doses. However, many aspects of the biological actions of these nanoscale cavitands remain unclear, including the immunotropic properties. In this study, we investigated the immunotoxicity and immunomodulation properties of CB[n]. It was found that CB[7] and CB[6] did not decrease the viability of mononuclear cells at all tested concentrations from 0.1–1 mM. Overall, the results indicated an immunomodulatory effect of different concentrations of CB[n]. In the case of a longer cultivation time, CB[n] had an immunostimulating effect, which was indicated by an enhancement of the proliferative activity of cells and increased expression of HLA-DR on lymphocytes.

## 1. Introduction

The use of nanoscale drug delivery systems is one of the most promising areas in modern pharmaceutical science [1,2]. Drug delivery systems are used for a variety of reasons, including to control the drug clearance; to protect the drug from biodegradation; for targeted delivery to specific organs, tissues, or cells; to reduce toxicity; and to increase solubility. However, before the application in clinical practice, nanostructures and nanomaterials used as components for such drug delivery systems require a comprehensive and careful study of their properties and biological activity. This research includes a study of the immunotropic properties of these substances because the cells of the immune system are the most sensitive to the damaging effect of nanomaterials.

One of the possible ways to create systems for the delivery of drugs is the use of nanoscale cavitands capable of host–guest complexation with drugs. Complexes of drugs can be obtained with various cavitands, including cyclodextrins, calixarenes, cucurbit[n]urils (CB[n]s), crown ethers, and cryptophanes. CB[n]s have a number of advantages, such as low toxicity, the ability to form stable complexes with various compounds, and the ability to bind with both hydrophobic and positively charged molecules. The formation of complexes of CB[n]s (*n* = 6, 7, and 8) with drugs can result in several benefits [3,4,5,6,7,8], including increasing the stability of the drug and reducing the rate of degradation in vivo, increased solubility, altered clearance, enabling a change in the route of administration (from parenteral to oral), and taste masking. The cavities of CB[6] and CB[7] (Figure S1 in Supplementary) are large enough to encapsulate transition metal complexes and small peptides [9]. Known examples of encapsulated drugs include cisplatin [10], oxaliplatin [11], and tuftsin [12]. However, to date, the effect of complexation with CB[n] on the biological properties of drugs is not completely clear.

Potential drug carriers should have low toxicity. In vitro and in vivo studies have shown that CB[n]s and their derivatives are inert and have practically no toxicity. At concentrations of up to 1 mM, CB[7] did not show cytotoxic activity toward various human and animal cell lines [13]. Moreover, the encapsulation of a drug into the cavity of CB[n] may reduce the toxicity of the drug [11,14,15]. Safety studies of CB[n] in vivo have also been performed [16,17,18,19,20,21,22]. Although CB[7] was found not hepatotoxic at 750 µM, it was cardiotoxic and affected motor activity at lower concentrations, and it was found even lethal after 48 hours, indicating a chronic toxicity [18].

When laboratory mice were administered CB[7], the maximum tolerated dose was 250 mg/kg. Intravenous administration of CB[n] is limited because of the low solubility, and not because of the development of side effects. For oral administration of a mixture of CB[6]–CB[8], the maximum tolerated dose was 600 mg/kg [17]. In another study of the biocompatibility of CB[7], Zhang et al. showed that a single oral dose of CB[7] at 5 g/kg did not lead to a significant decrease in animal body weight within 21 days after administration [22]. CB[7] has a dose-dependent toxicity. The administration of CB[7] at 750 mg/kg intraperitoneally and 200 mg/kg intravenously led to the rapid death of animals within a few minutes, while injections of 500 mg/kg peritoneally and 150 mg/kg intravenously did not result in any effects. Hematological tests, as well as tests for biochemical markers of hepatic and renal function, on blood taken from mice 21 days after the administration of CB[7] orally, intraperitoneally, and intravenously showed normal ranges of values, which were comparable to the control group. In addition, histological analysis of sections of the main organs (including the heart, liver, spleen, lungs, and kidneys) and the gastrointestinal tract revealed no visible injuries or signs of inflammation [22]. The biocompatibility of CB[6] only has not been studied.

Thus, the low toxicity and biomedical safety of CB[n]s have been demonstrated in various studies. However, to date, several aspects of the safety of CB[n]s remain unclear, including the effects on the immune system. It is important to determine the effect of nanoparticles on the immune system because the cells of the immune system are the most sensitive to the damaging effect of nanomaterials. In this study we investigated the in vitro immunotoxicity and immunomodulation properties of CB[6] and CB[7].

## 2. Results and Discussion

**Figure 1:**
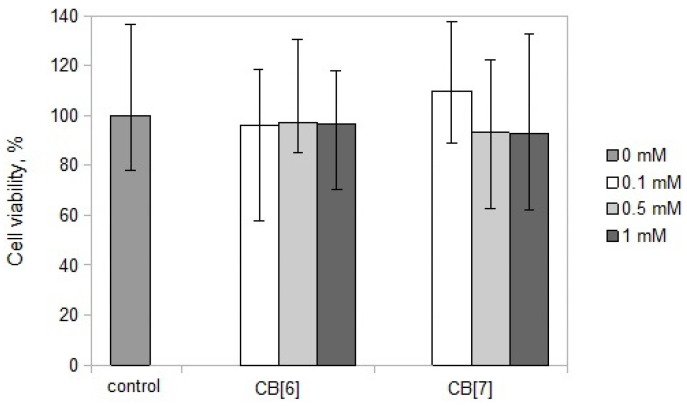
Viability of peripheral blood mononuclear cells (PBMCs) with or without cucurbit[n]uril (CB[n]) at different concentrations. Data are presented as the median with interquartile range.

CB[6]and CB[7] did not have a cytotoxic effect on PBMCs at all doses tested from 0.1 to 1 mM (Figure 1). The data obtained were in agreement with the literature data, which indicates that CB[n] is non-toxic in cells and human and animal tissues at micromolar concentrations [9,13]. Based on these data, we used concentrations of less than 1 mM for further experiments.

The next step was to study the effect of CB[n] on the relative amount of PBMCs in different phases of the cell cycle, including with spontaneous (Table 1) and anti-CD3 antibody-induced (Table 2) proliferation. Previously, we showed that CB[7] had no effect on the cell cycle, but only one concentration of CB[7] was used in this work [12]. In the present study, CB[6] and CB[7] did not affect the ratio of cells in different phases of the cell cycle and did not increase the relative number of sub-cycling hypodiploid cells in non-activated PBMCs at all tested concentrations. It was also shown that CB[6] and CB[7] did not affect the percentage of PBMCs in different phases of the cell cycle in cells activated by anti-CD3 antibodies and also did not lead to an increase in the relative number of hypodiploid cells. Therefore, CB[6] and CB[7], at the concentrations used, do not affect the percentage of PBMCs in different phases of the cell cycle.

**Figure 2:**
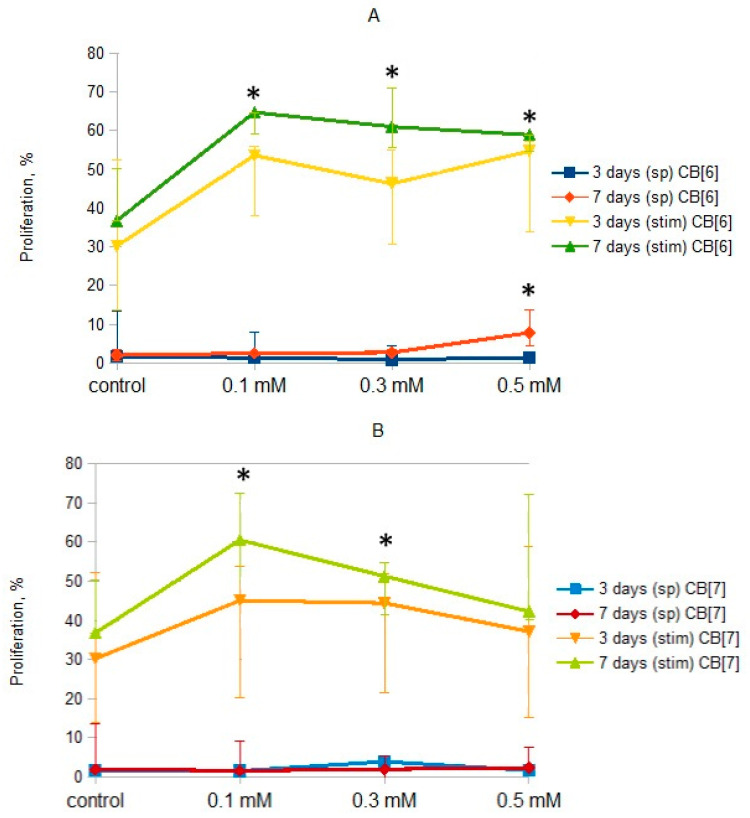
Effect of CB[n] on the relative number of proliferating T cells. (**A**): Effect of CB[6] on the relative number of proliferating T cells. (**B**): Effect of CB[7] on the relative number of proliferating T cells. Data are presented as the median with interquartile range. (* Indicates a significant difference (*p* < 0.05) vs. the control. (sp)—Spontaneous proliferation; (stim)—Anti-CD3-stimulated proliferation.

The effect of CB[n] on the proliferation of T-lymphocytes was evaluated on days 3 and 7. The study was performed both with and without stimulation of cell proliferation using anti-CD3 antibodies in combination with IL-2. It was found that CB[n] at all studied concentrations did not affect the proliferative activity of T-lymphocytes at day 3 (Figure 2). However, CB[6] enhanced both spontaneous and anti-CD3-induced proliferation of T-lymphocytes at day 7 of cultivation. CB[7] increased the stimulated proliferation of T-lymphocytes at concentrations of 0.1 and 0.3 mM, but not 0.5 mM. It is known that another cavitand, methyl-β-cyclodextrin, can also enhance the proliferation of PBMCs [23].

**Figure 3:**
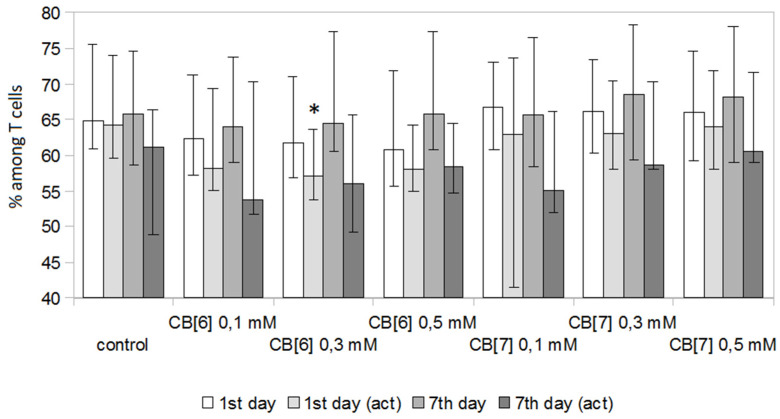
Percentage of CD3^+^CD4^+^ T-helper cells in PBMCs cultivated in the presence of CB[n] at various concentrations. Data are presented as the median with interquartile range. (* Indicates a significant difference (*p* < 0.05) vs. the control. (act)—Culture of PBMCs activated with anti-CD3 antibodies.

**Figure 4:**
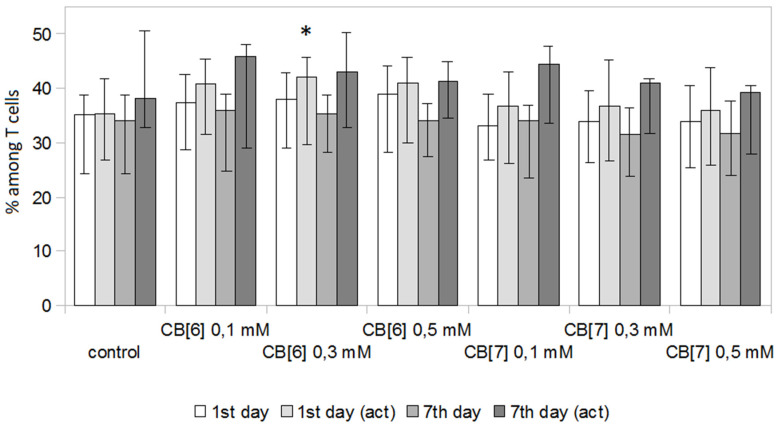
Percentage of CD3^+^CD4^−^ cytotoxic T cells in PBMCs cultivated in the presence of various concentrations of CB[n]. Data are presented as the median with interquartile range. (* Indicates a significant difference (*p* < 0.05) vs. the control. (act)—Culture of PBMCs activated with anti-CD3 antibodies.

The next step was to evaluate the effect of various concentrations of CB[n] on the percentage of T-helper cells and cytotoxic T-lymphocytes (Figure 3 and Figure 4). CB[n] had practically no effect on the number of T-helper cells and cytotoxic T-lymphocytes at days 1 and 7 of cultivation. However, 0.3 mM CB[6] in a stimulated culture led to a slight decrease in the number of T-helper cells (Figure 3) and a proportional increase in the number of cytotoxic T-lymphocytes (Figure 4) at day 1 of cultivation. Interestingly, this effect was not observed with further cultivation (day 7).

**Figure 5:**
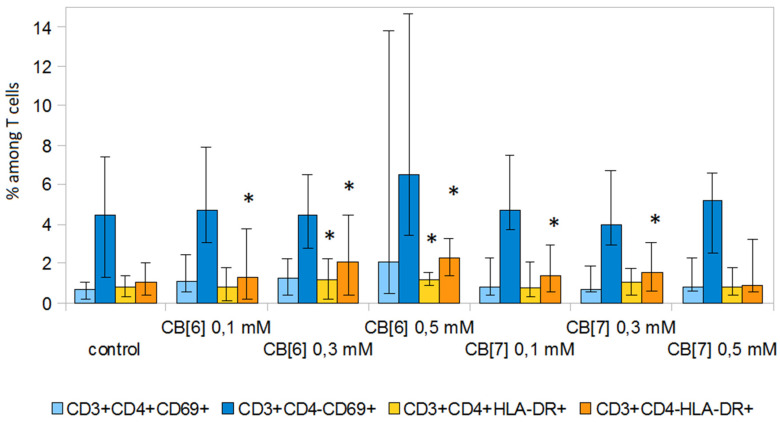
Effect of CB[n] on the expression of activation markers on T cells during the spontaneous proliferative activity of PBMCs. Data are presented as the median with interquartile range. (* Indicates a significant difference (*p* < 0.05) vs. the control).

**Figure 6:**
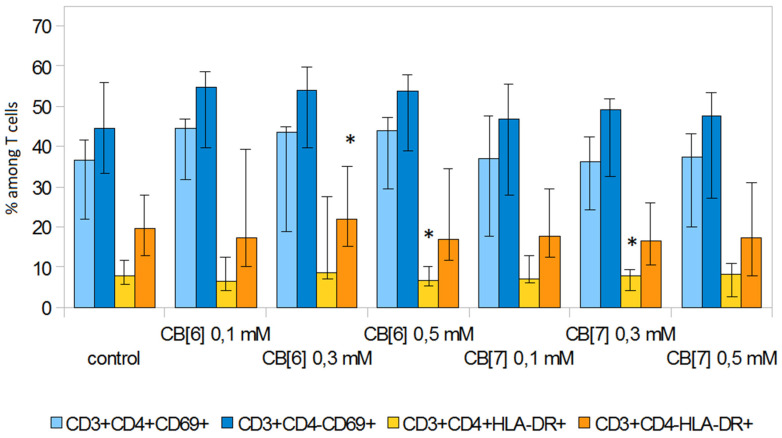
Effect of CB[n] on the expression of activation markers on T cells during anti-CD3-induced proliferative activity of PBMCs. (* Indicates a significant difference (*p* < 0.05) vs. the control).

In addition, we investigated the effect of CB[n] on early (CD69) and late (HLA-DR) markers of lymphocyte activation (Figure 5 and Figure 6). CB[n] did not lead to a change in the level of expression of CD69 on T-lymphocytes. However, it should be noted that significant differences (*p* < 0.05) in the expression of this molecule were observed in individual donors. CB[n] increased the expression of HLA-DR on CD3^+^CD4^−^ cells and decreased the expression of HLA-DR on CD3^+^CD4^+^ T lymphocytes in anti-CD3 stimulated culture. In addition, CB[n] increased the levels of HLA-DR on T-helper cells and cytotoxic T cells not activated with anti-CD3 antibodies.

**Figure 7:**
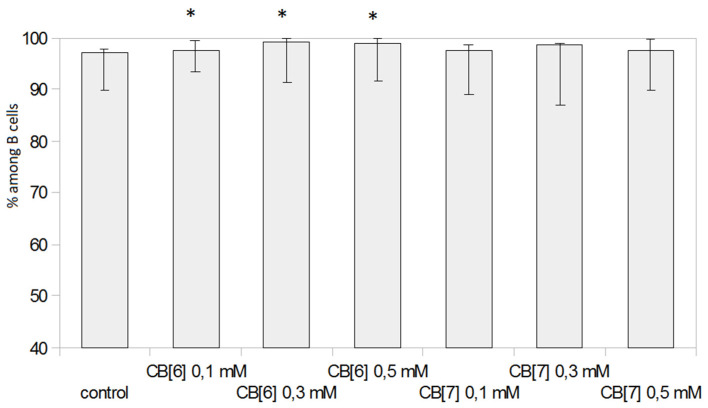
Effect of CB[n] on the HLA-DR expression on CD19+ B cells after seven days of cultivation. (* Indicates a significant difference (*p* < 0.05) vs. the control.)

Surprisingly, CB[6] at all studied doses increased the expression of HLA-DR on B lymphocytes after seven days of cultivation (Figure 7). These data indicate a possible effect of CB[6] on humoral immunity or the antigen presentation process. Other cavitands can also enhance the expression of CLA on antigen-presenting cells [24]. Thus, further research is required to identify the mechanism of enhancement of the HLA-DR expression.

## 3. Materials and Methods

### 3.1. Materials

CB[6] and CB[7] were synthesized at the Nikolaev Institute of Inorganic Chemistry SB RAS (Novosibirsk, Russia) through the standard procedure described in [25]. The structures of CB[6] and CB[7] were verified with ^1^H NMR in D_2_O at 25 °C (Figure S2 in Supplementary) using a 500 MHz Bruker Avance III spectrometer. Medium, phosphate-buffered saline, and L-glutamine were obtained from Biolot (Saint Petersburg, Russia). HyClone fetal calf serum was obtained from GE Healthcare (Chicago, IL, USA). WST-1 reagent was purchased from Takara Bio Inc. (Kusatsu, Japan). Anti-CD3 antibody was obtained from MedBioSpectr (Moskow, Russia). Recombinant IL-2 was obtained from Biotech (Saint Petersburg, Russia).

### 3.2. Isolation and Cultivation of Peripheral Blood Mononuclear Cells (PBMCs)

The venous blood of 22 healthy donors (mean age: 36.0 ± 2.48 years) was used in the study. Donor blood was obtained at the donor center of the State Budgetary Healthcare Institution of the Novosibirsk Oblast “City Clinical Hospital №1”. All the participants signed an informed consent form approved by the local ethics committee of the Research Institute of Fundamental and Clinical Immunology. PBMCs were isolated by the standard method by centrifugation of blood in a Ficoll–Urografin density gradient (ρ = 1.077 g/cm^3^) [26]. Cultivation of PBMCs was carried out in flat-bottom well plates (Costar, New York, NY, USA) at a concentration of 1 million/mL in Roswell Park Memorial Institute (RPMI-1640) (Biolot, St Petersburg, Russia) culture medium containing 0.3% L-glutamine (Biolot, St Petersburg, Russia), 50 μg/mL gentamicin (DalChimPharm, Russia), 25 μg/mL thienam (Merck Sharp & Dohme, Haarlem, The Netherlands), and 10% inactivated fetal calf serum (HyClone, Chicago, IL, USA) at 37 °C under 5% CO_2_ in air with high humidity.

### 3.3. Cell Viability

The cell viability was evaluated after incubation with different concentrations of CB[6] and CB[7] (1, 0.5, and 0.1 mM) using the WST-1 method. PBMCs were seeded at 10^5^ cells/well into a flat-bottomed, 96-well plate. After 72 h of cultivation, 10 μL of WST-1 (Takara Bio, Kusatsu, Japan) stock solution was added into each well containing 100 μL of cell suspension. The absorbance was directly read at 450 nm, and the reference was read at 620 nm.

### 3.4. Cell Cycle Analysis

Analysis of the PBMC cell cycle distribution was performed after 72 h of incubation with or without different concentrations of CB[n] (0.5, 0.3, and 0.1 mM). After cultivation, the PBMCs were washed from the medium with phosphate-buffered saline (Biolot, St Petersburg, Russia). Then, cells were fixed overnight at −20 °C in 70% ethanol and stained with propidium iodide (100 µg/mL) in the presence of RNAse A (100 µg/mL) for 30 min at 37 °C. Cell cycle analysis was conducted by evaluating DNA histograms. Samples were analyzed on a FACSCanto II flow cytometer (Becton Dickinson, Franklin Lakes, NJ, USA) and ModFit 3.2 software (Verity Software House, Topsham, ME, USA). The relative amounts of cells with diploid (cells in G_0_/G_1_ phases of the cell cycle) and hyperdiploid (cells in S and G_2_/M phases of the cell cycle) DNA sets were determined. Cells with fragmented DNA formed a characteristic hypodiploid peak.

### 3.5. T Cell Analysis

PBMCs were cultivated with different concentrations of CB[6] and CB[7] (0.5, 0.3, and 0.1 mM) in the presence or absence of anti-CD3 antibody (1 μg/mL) and recombinant human IL-2 (100 units/mL). The proliferation of PBMCs was assessed by flow cytometry of carboxyfluorescein succinimidyl ester (CFSE) stained cells. Cells were labeled before culture with 5,6-carboxyfluorescein diacetate succinimidyl ester (CFSE) (4 μM) (Invitrogen, Eugene, OR, USA), mixed well and incubated for 15 min in darkness, stirring occasionally. Then the reaction was stopped by adding 2 mL of PBS with 5% FCS, followed by centrifugation. To evaluate the proliferation of lymphocyte subsets, PBMCs after cultivation were stained with monoclonal anti-human antibodies (CD45-PE/Cy7, CD3-APC, CD4-PerCP/Cy5.5, and CD19-APC/Cy7) all from BioLegend, (San Diego, CA, USA). Analyses were performed using a FACSCanto II (Becton Dickinson, USA) and FACSDiva software (Becton Dickinson, USA). The analysis of proliferating activity was carried out after three and seven days of cultivation.

### 3.6. Expression of Activation Molecules

Cells were treated with CB[n] at different concentrations and cultured for 24 h or 1 w to evaluate early (CD69) and late (HLA-DR) activation markers, respectively. After cultivation, cells were stained with fluorochrome-labeled antibodies (CD69-PE or HLA-DR-PE, all from BioLegend).

### 3.7. Statistical Analysis

All data from experiments were expressed as median (25th–75th percentile). ANOVA analyses were performed using GraphPad Prism, with post-hoc comparisons carried out by Fisher’s protected least significant difference tests. A *p*-value < 0.05 was regarded as the minimum criterion for statistical significance.

## 4. Conclusions

Our results showed that CB[n] (*n* = 6 and 7) did not affect the viability of PBMCs. In the case of a short cultivation time (1–3 days), CB[n] did not have an appreciable effect on the proliferation and phenotypic characteristics of the PBMCs. After a longer cultivation time, CB[n] had an immunostimulating effect, enhancing the proliferative activity of cells and increasing the expression of HLA-DR on lymphocytes. It is important to note that CB[n] did not have an immunosuppressive effect, with the exception of a slight decrease in HLA-DR expression on T-helper cells in stimulated cultures.

## Figures and Tables

**Table 1:** Relative number of cells in different phases of the cell cycle during the spontaneous proliferative activity of PBMCs cultured for 72 h in the presence of various concentrations of CB[n]. Data are presented as the median with interquartile range.

	Sub-G_1_/G_0_	G_1_/G_0_	S	G_2_
Control	0.6 (0.4–1.6)	98.7 (95.7–99.3)	0.6 (0.3–2.5)	0.2 (0.1–0.4)
CB[6] 0.1 mM	1.0 (0.3–2.0)	97.9 (96.7–98.9)	0.8 (0.6–1.4)	0.1 (0.1–0.2)
CB[6] 0.3 mM	1.1 (0.4–5.0)	97.2 (88.6–97.9)	1.3 (1.0–6.1)	0.6 (0.3–0.8)
CB[6] 0.5 mM	0.6 (0.3–11.7)	97.7(79.8–98.6)	1.3 (1.1–7.7)	0.5 (0.2–1.0)
CB[7] 0.1 mM	0.2 (0.1–0.3)	99.2 (99.0–99.5)	0.3 (0.3–0.6)	0.1 (0.1–0.3)
CB[7] 0.3 mM	1.2 (0.2–6.4)	96.6 (91.0–98.6)	1.9 (1.1–2.4)	0.2 (0.2–0.3)
CB[7] 0.5 mM	1.2 (0.3–2.4)	98.0 (93.1–99.2)	0.7 (0.5–4.1)	0.2 (0.1–0.3)

**Table 2:** Relative number of cells in different phases of the cell cycle during anti-CD3-induced proliferative activity of PBMCs cultured for 72 h in the presence of different concentrations of CB[n]. Data are presented as the median with interquartile range.

	Sub-G_1_/G_0_	G_1_/G_0_	S	G_2_
Control	2.8 (1.3–5.7)	56.4 (55.1–69.6)	30.4 (20.6–31.9)	9.5 (7.8–10.1)
CB[6] 0.1 mM	4.4 (2.3–5.4)	58.6 (55.5–72.7)	27.8 (17.7–31.7)	8.4 (6.4–9.2)
CB[6] 0.3 mM	3.3 (2.0–4.6)	58.4 (52.5–74.4)	28.0 (16.9–33.4)	9.1 (5.4–10.9)
CB[6] 0.5 mM	3.5 (2.0–4.5)	58.5 (52.9–72.5)	28.7 (19.0–33.1)	8.6 (6.5–9.7)
CB[7] 0.1 mM	2.3 (1.1–3.5)	61.3 (54.1–76.8)	28.1 (16.5–31.7)	8.3 (5.3–11.2)
CB[7] 0.3 mM	2.8 (1.4–3.5)	59.6 (54.0–74.1)	28.2 (18.2–32.1)	8.8 (6.1–10.7)
CB[7] 0.5 mM	3.9 (1.7–5.4)	58.8 (56.5–75.6)	27.1 (16.3–29.7)	8.1 (5.1–9.6)

## Supplementary Materials

The Supplementary Materials are available online.
