# Supplementary material for: Evaluation of the Immunosafety of Cucurbit[n]uril on Peripheral Blood Mononuclear Cells In Vitro
Source: Molecules. 2020 Jul 27;25(15):3388. doi: 10.3390/molecules25153388 (PMC7435832; doi:10.3390/molecules25153388)
Supplement: Supplementary file 1 [file molecules-25-03388-s001.pdf]

Supplementary materials

Evaluation of the Immunosafety of Cucurbit[n]uril on Peripheral Blood Mononuclear Cells In Vitro

Ekaterina Pashkina[1,2,4], Alina Aktanova[1,4], Elena Blinova[1], Irina Mirzaeva[3], Ekaterina Kovalenko[3], Nadezhda Knauer[1,4], Aleksandr Ermakov[2] and Vladimir Kozlov[1,2]

[1] Research Institute of Fundamental and Clinical Immunology, 14, Yadrintsevskaya st., 630099 Novosibirsk, Russia. E-mail: pashkina.e.a@yandex.ru (E.P.), aktanova_al@mail.ru (A.A), blinovaelena-85@yandex.ru (E.B.), knauern@gmail.com (N.K.), niiki01@online.nsk.su (V.K.).

[2] Novosibirsk State Medical University, 52, Krasny Prospect, 630091 Novosibirsk, Russia. E-mail: aleermak@mail.ru (A.E.).

[3] Nikolaev Institute of Inorganic Chemistry SB RAS, 3, Lavrentiev ave., 630090 Novosibirsk, Russia. E-mail: dairdre@gmail.com (I.M.), e.a.kovalenko@niic.nsc.ru (E.K.).

[4] Institute of Chemical Biology and Fundamental Medicine SB RAS, 8, Lavrentiev ave., 630090 Novosibirsk, Russia.

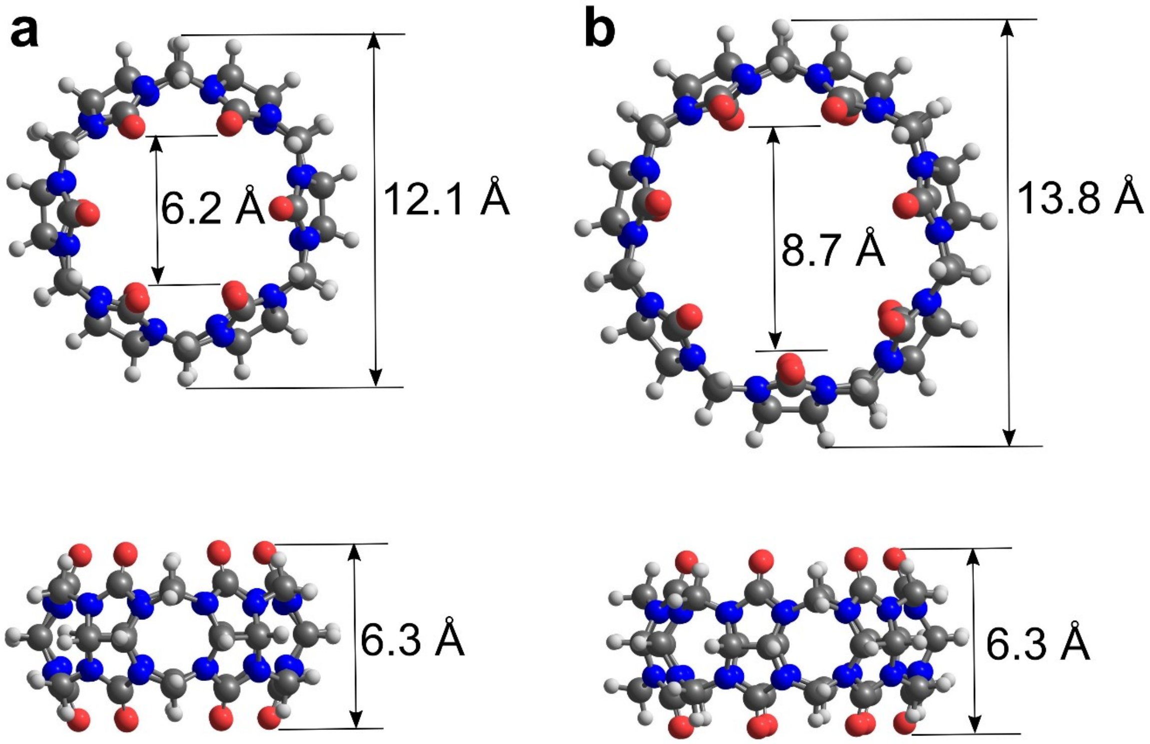

Figure S1. Top and side views of the structures of a – CB[6] and b – CB[7]. The structures were optimized in ADF2017 program suit (www.scm.com) with BLYP+D3(BJ)/TZP level of theory.

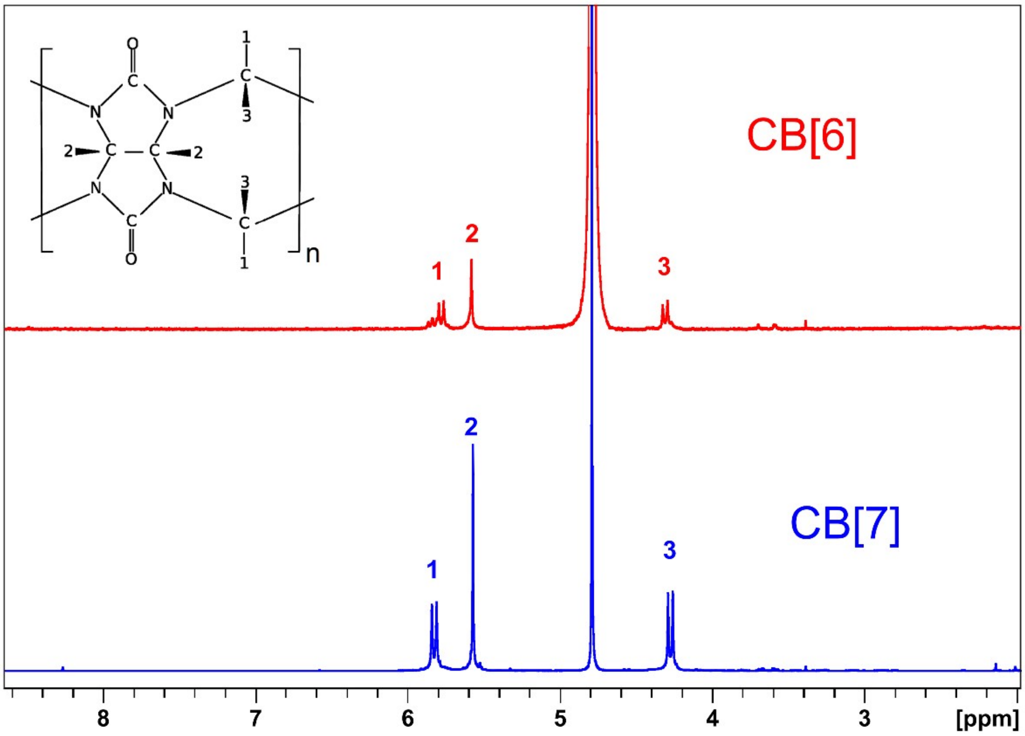

Figure S2. ^1H NMR spectra of CB[6] and CB[7] in D_2O at 25 ºC.

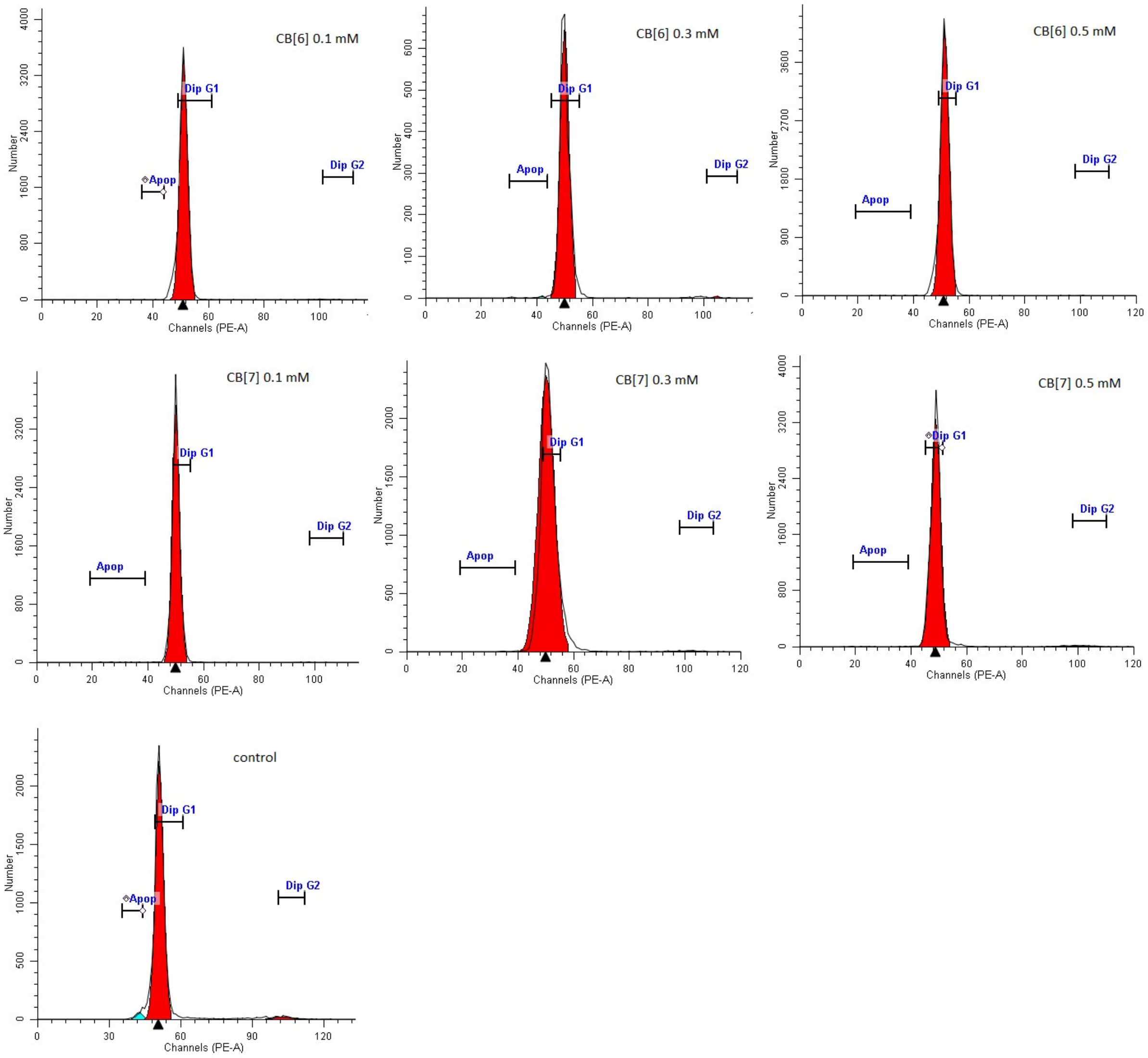

Figure S3a. Cell cycle distribution in non-activated PBMCs cultured for 72 h in the presence of various concentrations of CB[n].

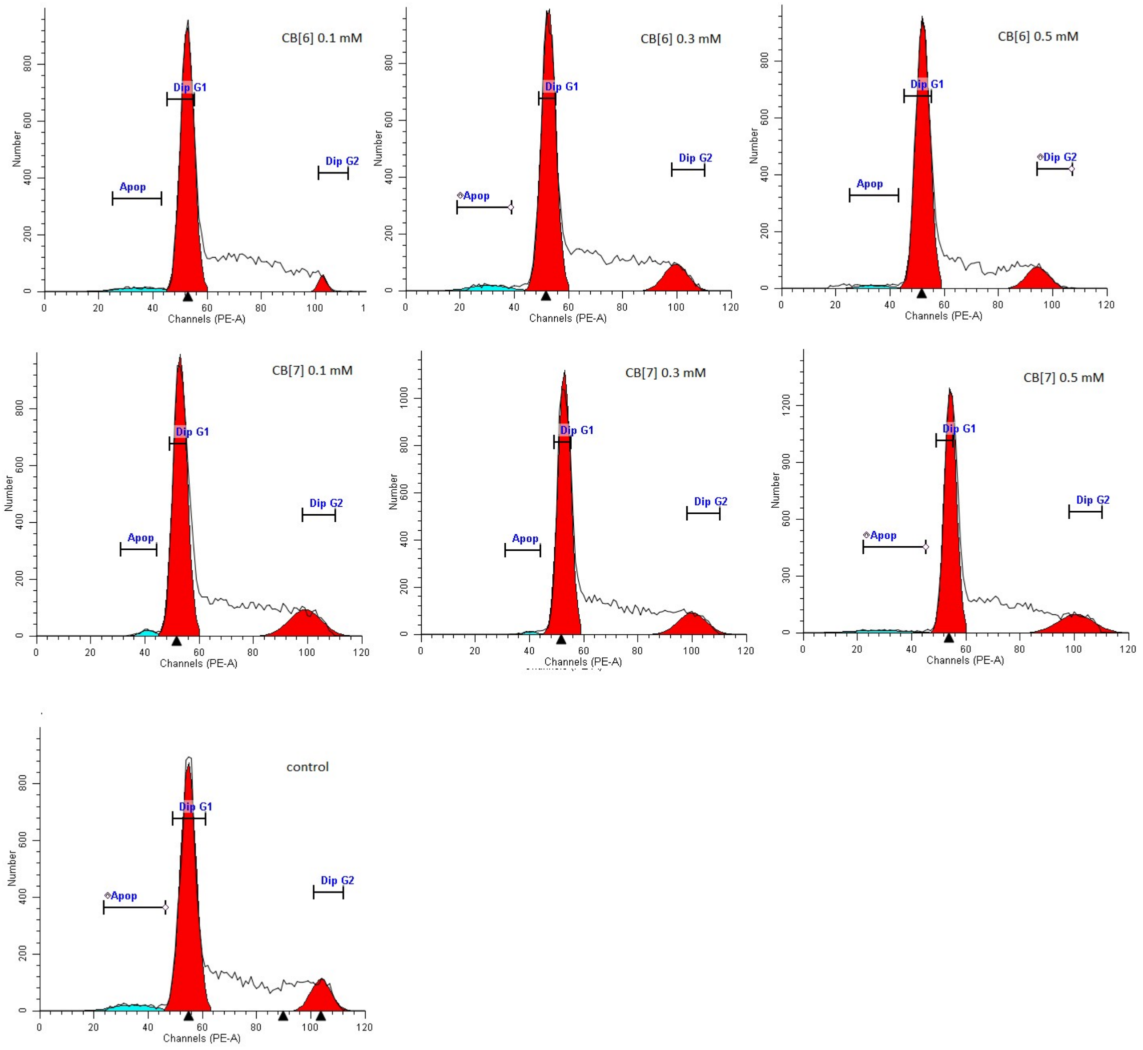

Figure S3b. Cell cycle distribution in aCD3-activated PBMCs cultured for 72 h in the presence of various concentrations of CB[n].
